# Non-Alcoholic Fatty Liver Disease and COVID-19–Two Pandemics Hitting at the Same Time

**DOI:** 10.3390/medicina57101057

**Published:** 2021-10-03

**Authors:** Luka Vranić, Anja Radovan, Goran Poropat, Ivana Mikolašević, Sandra Milić

**Affiliations:** Department of Gastroenterology and Hepatology, University Hospital Centre Rijeka, School of Medicine, University of Rijeka, 51000 Rijeka, Croatia; anja.radovan@uniri.hr (A.R.); goran.poropat@uniri.hr (G.P.); ivana.mikolasevic@medri.uniri.hr (I.M.)

**Keywords:** COVID-19, liver injury, metabolic-associated fatty liver disease, metabolic syndrome, non-alcoholic fatty liver disease

## Abstract

The COVID-19 pandemic was and still is a global burden with more than 178,000,000 cases reported so far. Although it mainly affects respiratory organs, COVID-19 has many extrapulmonary manifestations, including, among other things, liver injury. Many hypotheses have been proposed to explain direct and indirect impacts of the SARS-CoV-2 virus on the liver. Studies have shown that around 15–30% of patients with COVID-19 have underlying liver disease, and 20–35% of patients with COVID-19 had altered liver enzymes at admission. One of the hypotheses is reactivation of an underlying liver disease, such as non-alcoholic fatty liver disease (NAFLD). Some studies have shown that NAFLD is associated with severe COVID-19 and poor outcome; nevertheless, other studies showed no significant difference between groups in comparing complications and clinical outcomes. Patients with NAFLD may suffer severe COVID-19 due to other comorbidities, especially cardiovascular diseases. The link between NAFLD and COVID-19 is not clear yet, and further studies and research are needed.

## 1. Introduction

The pandemic of severe acute respiratory syndrome (SARS) caused by the coronavirus SARS-CoV-2 (COVID-19, Sarbecovirus subgenus, Betacoronavirus genus, Coronaviridae family) started in the city of Wuhan, Hubei province, China, in December 2019. Soon after, it became a global burden as the pandemic progressed. By June 2021 more than 178,000,000 cases have been reported, as well as 3,860,000 deaths [1,2].

Even though the majority of subjects remain asymptomatic, acting as a primarily respiratory virus, it mainly affects respiratory organs, causing symptoms from cough, fever, nasal congestion, fatigue, loss of smell and taste, and flu like symptoms, up to severe pneumonia and acute respiratory distress syndrome (ARDS). However, we have learned that COVID-19 is actually a systemic disease, meaning that extrapulmonary manifestations, such as thrombotic incidents, gastrointestinal symptoms, cardiac symptoms and arrythmias, and ocular or neurological symptoms are not uncommon [3]. Liver injury in COVID-19 patients has been observed with an incidence from 14% to 53% [4,5]. However, major clinically significant liver damage is infrequent [6]. 

Starting long before the COVID-19 pandemic, in the last few decades, a continuously increasing incidence of obesity and metabolic syndrome (MetS) has become a major public health problem. Non-alcoholic fatty liver disease (NAFLD) is, in fact, a metabolic liver disease directly linked to MetS and its individual conditions [7]. It has been estimated that the prevalence of NAFLD is from 20–30%, varying from country to country [8]. It is predicted that by 2030, NAFLD is going to be the leading cause of liver cirrhosis and the most frequent indication for liver transplantation [9]. With our continuously increasing knowledge of NAFLD pathogenesis, we understand that NAFLD represents a state of metabolic dysregulation and chronic low-grade inflammation alongside other conditions of MetS [7]. Thus, recently a new acronym MAFLD (metabolic-associated fatty liver disease) has been suggested and used because it emphasizes the role of metabolic dysregulation in NAFLD pathogenesis [10]. 

Being the most prevalent chronic liver disease, it is obvious that during SARS-CoV-2 outbreak, a lot of people suffer from both conditions simultaneously. However, the true interaction between these two pandemics is still not fully understood. Because of high-lethality of COVID-19 and global repercussions of outbreak, better understanding of virus behavior, risk factors of COVID-19 disease progression and its interaction with other frequent diseases is mandatory for anticipation of virus-related events worldwide [11].

In present review, we discuss the interaction of two different pandemics: a few decades old NAFLD pandemic and the recent SARS-CoV-2 outbreak. Theoretically, in COVID-19 patients, NAFLD and associated metabolic dysregulation and chronic low-grade inflammation could pave a path to more serious liver and systemic complications and could also serve as a prognostic marker of ongoing viral disease [12,13].

## 2. Mechanisms of Liver Injury in COVID-19 

It has been documented that people with liver diseases, especially liver cirrhosis and hepatocellular carcinoma, are more susceptible to infections and sepsis due to impaired immunity [14,15]. Therefore, it is understandable that NAFLD could predispose to more severe COVID-19. Moreover, MetS increases the risk for cardiovascular diseases, which has also been identified as a risk factor for higher mortality in COVID-19 [16].

The pathogenic properties of SARS-CoV-2 depend on binding of spike viral proteins to angiotensin I converting enzyme 2 (ACE2) receptors which allows the virus to enter target cells and replicate [17,18]. These receptors are expressed predominantly in the mucosal cells of the upper respiratory tract, in alveolar cells type II and ciliated cells [19]. These cells represent its major site of replication. Moreover, ACE2 receptors have been found in vascular endothelium, renal proximal tubule cells, stomach enterocytes, brush border of enterocytes, colonocytes and cholangiocytes [18], making symptomatic involvement of all these organs possible. A case-control study from USA reported gastrointestinal symptoms present in 61% of COVID-19 patients [20]. The presence of ACE2 receptors along gastrointestinal tract can result in dysregulation of the enteric nervous system, malabsorption and uncontrolled secretion, leading to various symptoms [11,21,22].

The liver can be affected by COVID-19, but major liver damage is very uncommon [6,11,23]. Hepatic ACE2 receptors are mainly expressed in cholangiocytes and endothelial cells (overall 60% of cells). Only 3% of hepatocytes express these receptors and none are present in macrophages (Kupffer cells) [11,24]. 

**Figure 1:**
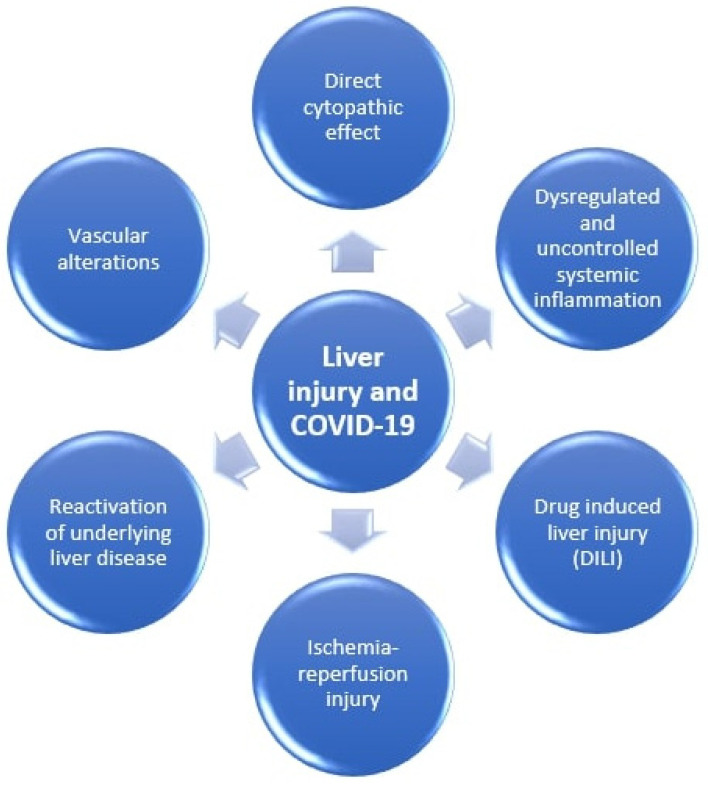
Mechanisms of liver injury in COVID-19.

There have been several mechanisms proposed that lead to liver damage including direct cytopathic effect of the virus or indirect damage caused by inducing systemic inflammation, liver ischemia and hypoxia, drug-related liver injury and exacerbation of pre-existing liver disorders [11,25]. These mechanisms are graphically shown in Figure 1. 

Direct cytopathic effect of the virus could be due to viral replication in cholangiocytes that express ACE2 receptors and consequent bile duct epithelial cell damage. However, significant elevation of cholestatic enzymes (that reflect bile duct injury) is rarely reported in COVID-19 patients [12]. Liver histopathological findings of COVID-19 patients did not show significant damage of hepatocytes, vascular cells nor bile duct cells [26]. Moreover, the most common pre-existing liver disorder, NAFLD, is not associated with altered liver expression of SARS-COV 2 critical entry points including ACE2 receptors [27]. Therefore, it is fair to speculate that liver injury is more likely to be due to secondary liver damage. 

Dysregulated and uncontrolled systemic inflammation is the most probable cause of liver injury in COVID-19 patients. Although disease itself is usually mild, a minority of patients present with severe clinical features. In the background of severe COVID-19 cases lie a systemic inflammatory response syndrome (SIRS), an uncontrolled cytokine “storm” that is caused by unregulated activation of both natural and cellular immunity. It causes multiple organ injuries and failures including the liver [12]. It appears that IL-6 is one of the key factors in the onset and progression of cytokine storm in COVID-19 patients. Therefore, tocilizumab, IL-6 monoclonal antibody has been approved for treatment of severe COVID-19 episodes. This cytokine is also a link between COVID-19 and dysregulated metabolic conditions including NAFLD/MAFLD. A population-based, observational cohort of 6814 people without previously known cardiovascular disease (CVD) showed that IL-6 was significantly more prevalent in NAFLD patients, and that it was the only biomarker that could predict subclinical CVD determined by non-contrast cardiac CT [28].

How NAFLD could contribute to liver injury in COVID-19 patients or any other severe form of systemic inflammation is not completely understood. However, it might be related to impaired innate immunity to the virus. Recently published research showed that obesity, undoubtedly related to NAFLD, promotes the shift of inflammation-suppressing M2 macrophages to pro-inflammatory M1 macrophages. This unique polarization of macrophages is caused by fatty acids which leads to ectopic lipid accumulation and local and systemic chronic low-grade inflammation [29]. This metabolic and immunological dysregulation could exacerbate SARS-CoV-2 infection leading to more severe COVID-19 disease.

Ischemia-reperfusion injury during respiratory failure or sepsis is mostly complementary with previously mentioned dysregulated and uncontrolled immune system. In case of severe COVID-19 presentation when systemic inflammatory response syndrome (SIRS) occurs, uncontrolled release of proinflammatory cytokines causes vasodilatation of peripheral vessels which lead to low blood pressure and generalized tissue hypoxia. If acute respiratory distress syndrome (ARDS) occurs simultaneously, it leads to poor blood oxygenation that aggravates liver ischemia alongside already poor blood supply. Under shock and hypoxia, reactive oxygen species (ROS) increase which leads to enhanced peroxidation of lipids, DNA and proteins. These peroxidation products and ROS itself activate hepatic stellate cells to produce extracellular matrix and innate liver immune cells that produce proinflammatory cytokines leading to further liver damage [30,31]. 

Drug induced liver injury (DILI) can sometimes occur in COVID-19 patients. In cases of severe episodes, patients are being treated with large number of different medications, treating both COVID-19 and underlying (possibly worsening) comorbidities. Interaction between lot of different medications could be hepatotoxic. Antipyretic drugs, such as acetaminophen, in large doses can be hepatotoxic, especially in patients with a pre-existing liver disorder. Moreover, even though targeted antiviral agents for SARS-COV 2 have not yet been introduced, several antiviral or monoclonal antibodies have been used, including remdesivir, lopinavir/ritonavir, oseltamivir or tocilizumab that can potentially be hepatotoxic in some patients. Other drugs such as corticosteroids, carbapenems, azithromycin, hydroxychloroquine, fluconazole or ketoconazole and propofol can also cause liver injury. Several authors hypothesize that presence of underlying metabolic disorders and NAFLD/MAFLD in COVID-19 patients can precipitate DILI. Hypotheses are being based on assumptions that pre-existing metabolic liver disorders can exaggerate hepatotoxicity of drugs due to impaired activity of metabolizing enzymes, oxidative stress, mitochondrial dysfunction and altered lipid homeostasis. On the other hand, some xenobiotics might lead to worsening of NAFLD, meaning triggering transition of fatty liver to nonalcoholic steatohepatitis (NASH) and thereby inducing necroinflammation and consequently fibrosis and rapid development of liver cirrhosis [32,33]. 

Reactivation of underlying liver disease can be a cause of liver injury in COVID-19. Studies have shown that around 15–30% of patients with COVID-19 have underlying liver disease, and 20–35% of patients with COVID-19 have altered liver enzymes at admission [34,35]. Hashemi et al. showed in their study that the prevalence of elevated liver enzymes was higher in patients with underlying liver disease, as well as higher need for mechanical ventilation, higher rates of ICU stay and all-cause mortality. When they stratified the patients with underlying liver injury into two groups, NAFLD and other etiologies, significantly higher rates of ICU admission and need for mechanical ventilation were shown in patients with NAFLD [36]. 

Immunosuppressive drugs used in treatment of severe COVID-19, such as corticosteroids, IL-1 receptor antagonists, IL-6 receptor antagonists and JAK inhibitors can potentially cause hepatitis B reactivation. However, studies have shown that the risk of reactivation is moderate to low. Current guidelines recommend screening for HBsAg and anti-HBc before the application of immunosuppressive therapy. All patients at moderate to high risk of hepatitis B reactivation should receive prophylactic anti-HBV therapy [37,38,39].

Vascular alterations are another possible cause of liver injury. This is supported by the study of Sonzogni et al. that analyzed liver samples of patients who died from COVID-19 due to respiratory failure and observed minimal inflammatory infiltrate. Partial or complete luminal thrombosis of portal and sinusoidal vessels, portal tract fibrosis and dilatation of portal vein branches were noticed; however, these were probably secondary due to impaired circulation within the liver and coagulation cascade induced by the virus [40]. On the other hand, another post-mortem study in COVID-19 patients showed fatty change of the liver in 88% of patients with no liver thrombosis; however, the sample size was small [41].

## 3. Interaction between NAFLD/MAFLD and COVID-19

Numerous observational studies show that patients with comorbidities such as cardiovascular disease, arterial hypertension, diabetes mellitus, chronic lung diseases or cancer are more prone to more severe COVID-19 episodes [42,43]. Some of those conditions, alone or in combination, predispose to accumulation of fat in liver (NAFLD) and associated metabolic changes. Liver impairment has been commonly observed among COVID-19 patients [13,34,35]. Despite being primarily a respiratory disease, transaminemia has been related to higher mortality in COVID-19 patients [44]. A study by Chen et al. reported that alanine aminotransferase (ALT) and aspartate aminotransferase (AST) were increased in 28% and 35% of COVID-19 patients, respectively [34]. Guan et al. analyzed clinical characteristics of COVID-19 patients and found out that 21.3% and 22.2% of patients had elevated ALT and AST, respectively [35]. These studies suggest that liver involvement is quite common in COVID-19. 

Chronic liver diseases (CLD) represent a great disease burden globally. Given the high burden and still uncontrolled COVID-19 pandemic with increasing number of patients daily, how these CLDs influence further liver injury, disease course and mortality in COVID-19 patients has to be thoroughly evaluated. These conditions are alcohol-related liver disease, NAFLD, chronic viral hepatitis and other rarer conditions. It has been observed that 2–11% of COVID-19 patients have CLD comorbidity [13]. Being the most prevalent CLD, NAFLD often emerges as a comorbidity among COVID-19 patients [45]. 

Clinical features of patients with both conditions are still unclear. Recently, several meta-analyses have demonstrated that obesity and diabetes (both highly related to NAFLD) were significantly associated with more severe disease course and increased mortality in COVID-19 [46,47]. There has been a clear correlation between excess body fat and autoimmune disease with susceptibility to infections, which confirms assumptions that overweight people have impaired and altered immune responses. Viral respiratory infections are also more prevalent in obese people compared to population with normal body weight [11,48]. Zheng et al. reported a six-fold increased risk of severe COVID-19 in the presence of obesity in NAFLD patients in the logistic regression model even after adjusting for age, sex, smoking, diabetes, hypertension, and dyslipidaemia [49]. Moreover, a meta-analysis showed that obesity can aggravate COVID-19 infections, meaning that body mass index (BMI) is higher in severe COVID-19 patients and that obesity was associated with poor outcomes in terms of severe COVID-19, disease progression, ICU care and use of mechanical ventilation. The risk for severe COVID-19 in obese patients was greater in younger patients. However, obesity did not increase the risk of hospital mortality [50]. 

In a multicenter retrospective study with 280 patients, Huang et al. investigated clinical features of COVID-19 patients with special attention to NAFLD patients. NAFLD was present in 30.7% of patients, which is consistent with the number from the general population. Most common symptoms were fever (66.8%) and cough (55.7%) followed by fatigue, sore throat, shortness of breath, muscle ache and headache. However, the frequency of symptoms was not significantly different between NAFLD and non-NAFLD patients. NAFLD patients had higher median levels of ALT and GGT at admission, as well as higher peak of ALT and GGT during hospitalization confirmed by logistic regression. This suggests that NAFLD patients have higher risk to develop liver injury, although liver enzyme levels were not generally high at admission or during hospitalization. Severe liver damage was not observed. Moreover, NAFLD was not associated with adverse clinical outcomes in COVID-19 patients, and patients were generally young with a low proportion of comorbidities [45]. Another study revealed that NAFLD was significantly more prevalent in severe COVID-19 patients than stable patients (87.2% vs. 25.8%); however, median age and number of comorbidities were also significantly higher in severe COVID-19 patients. NAFLD patients had significantly higher risk of disease progression, likelihood of alteration of liver enzymes and longer viral shedding time compared to non-NALFD patients [4]. In a series of 310 COVID-19 and NAFLD patients, Targher et al. showed that intermediate of high FIB-4 and NFS scores greatly and independently correlate with severe COVID-19 disease progression [51]. 

On the other hand, study by Zhou et al. revealed that NAFLD/MAFLD was significantly associated with severe cases only in patients younger than 60 years [52]. Moreover, Mushtaq et al. found that NAFLD is an independent predictor of mild to moderate liver injury but was not associated with important clinical outcomes such as mortality, disease severity at presentation nor disease progression in patients with COVID-19 [53]. A list and details of mentioned primary studies investigating relation between NAFLD and COVID-19 are provided in Table 1.

A systematic review evaluated specifically NAFLD as risk factor in COVID-19 patients and revealed pooled OR for severe COVID-19 in NAFLD, after adjusting for obesity, of 2.358 (95% CI: 1.902–2.923, *p* < 0.001) [14]. This indicates that fat accumulation itself, without confounding factors, could exacerbate virus-associated cytokine storm that leads to worse COVID-19 outcomes, even though the exact cause remains to be elucidated. A meta-analysis that included 1293 patients from six retrospective studies showed that MAFLD increased the risk of severe COVID-19 with pooled OR of 2.93; however, this was not statistically significant (*p* = 0.166) [54]. Thus, the real impact of NAFLD in COVID-19 patients is something that needs to be further investigated.

## 4. Conclusions

Liver involvement in both NAFLD and COVID-19 patients seems to be only mild to moderate, which questions its clinical importance in COVID-19. On the other hand, there are conflicting data regarding susceptibility to more severe COVID-19 and disease progression in NAFLD patients. It is not clear if NAFLD patients may suffer from more severe COVID-19 due to NAFLD per se or due to other comorbidities that frequently come along NAFLD, especially cardiovascular diseases. Since COVID-19 is a new disease, more prospective or larger retrospective studies are needed in order to better understand virus behavior and its interaction with other illnesses.

## Figures and Tables

**Table 1:** Studies that investigated the connection between NAFLD and COVID-19.

Ref	Study Design	Sample Size	Major Findings
Zheng et al. [49]	Multicentric, prospective study	66	>6-fold increased risk of severe COVID-19 in the presence of obesity in NAFLD patients
Huang et al. [45]	Multicentric, retrospective study	280	NAFLD was not associated with adverse clinical outcome in COVID-19 patients, but patients were generally young and proportion of comorbidities was low
Ji et al. [4]	Retrospective study	202	NAFLD patients had significantly higher risk of disease progression, likelihood of alteration of liver enzymes and longer viral shedding time
Targher et al. [51]	Multicentric, prospective study	310	Intermediate of high FIB-4 and NFS scores greatly and independently correlates with severe COVID-19 disease progression
Zhou et al. [52]	Multicentric, retrospective study	327	NAFLD/MAFLD was significantly associated with severe cases only in patients younger than 60 years
Mushtaq et al. [53]	Retrospective study	589	NAFLD is independent predictor of mild to moderate liver injury, but not associated with important clinical outcomes in patients with COVID-19

## Data Availability

Data is contained within the article.
